# Acute Acalculous Cholecystitis Due to COVID-19, an Unusual Presentation

**DOI:** 10.7759/cureus.15431

**Published:** 2021-06-03

**Authors:** Fuad I Abaleka, Bisrat Nigussie, Genanew Bedanie, Amir Mohammed, Selin Galiboglu

**Affiliations:** 1 Internal Medicine, Richmond University Medical Center, Staten Island, USA; 2 Internal Medicine, Texas Tech University Health Sciences Center, Lubbock, USA; 3 Internal Medicine, Wellstar Atlanta Medical Center, Atlanta, USA

**Keywords:** covid 19, acalculus cholecystitis, acute cholecystitis, covid 19 gi manifestation, covid 19 pneumonia

## Abstract

Although the Coronavirus Disease 2019 (COVID-19) infection mainly affects the lung, its gastrointestinal (GI) involvements are also well-known, especially hepatic involvement presenting as mild to moderate transaminitis. However, COVID-19 infection presenting with gall bladder involvement with acalculous cholecystitis is extremely rare in the medical literature. So far, only two cases have been reported, and herein, we are reporting the third case of a patient who developed COVID-19 presenting as an acute acalculous cholecystitis.

## Introduction

The common presentation of Coronavirus Disease 2019 (COVID-19) viral infection includes fever, myalgia, and respiratory symptoms such as dry cough, shortness of breath, and loss of smell [1]. COVID-19 is caused by severe acute respiratory syndrome coronavirus 2 (SARS-CoV-2). Besides lung involvement, COVID-19 is also known to affect the liver. Most recent studies showed that β-coronaviruses from lineage B that are highly pathogenic to humans, such as the SARS-CoV (2002) and SARS-CoV-2 (2019), can affect the liver and induce acute hepatitis [1,2]. However, COVID-19 gall bladder involvement is extremely rare. Herein, we report a case of COVID-19 presenting as acute acalculous cholecystitis.

## Case presentation

A 76-year-old female with a medical history of atrial fibrillation, congestive heart failure (CHF) on pacemaker, hypertension, and asthma presented to the emergency department with the chief complaint of right upper quadrant abdominal pain of seven days duration. The patient described the pain as gradual in onset, which progressively worsened over two days duration. The pain was dull in quality, radiated to her right shoulder, worsened with meals, taking a deep breath, and moving. She described the severity of pain as 8/10; it was associated with nausea and vomiting of ingested matter for the past three days. She denied any alleviating factors. Furthermore, the patient complained of dry cough and gradual worsening of shortness of breath which started seven days ago. Shortness of breath was both at rest and while walking. The patient also had intermittent low-grade fever with a T-max of 100.8°F in the past three days. Otherwise, she denied chest pain, leg swelling, hemoptysis, yellowish discoloration of the eye, change in stool color, diarrhea, or constipation. 

**Figure 1:**
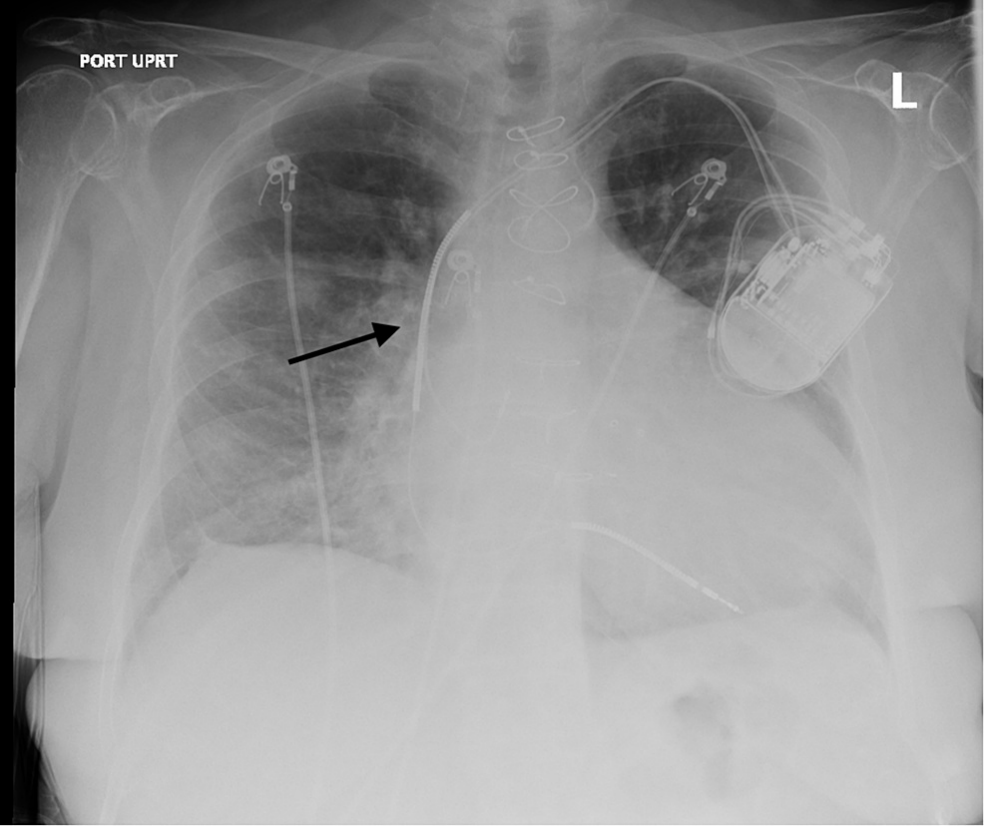
Chest X-ray: arrow showing interstitial infiltrates in the right lung

**Figure 2:**
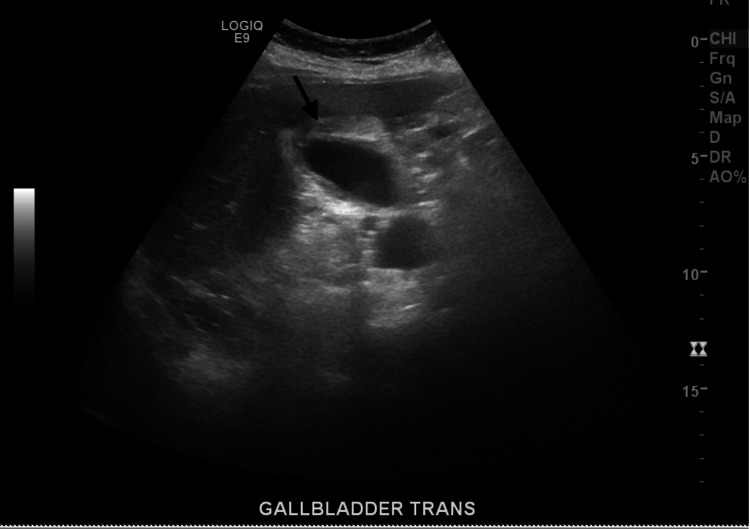
Ultrasound of gallbladder: arrow showing pericholecystic fluid collection

**Figure 3:**
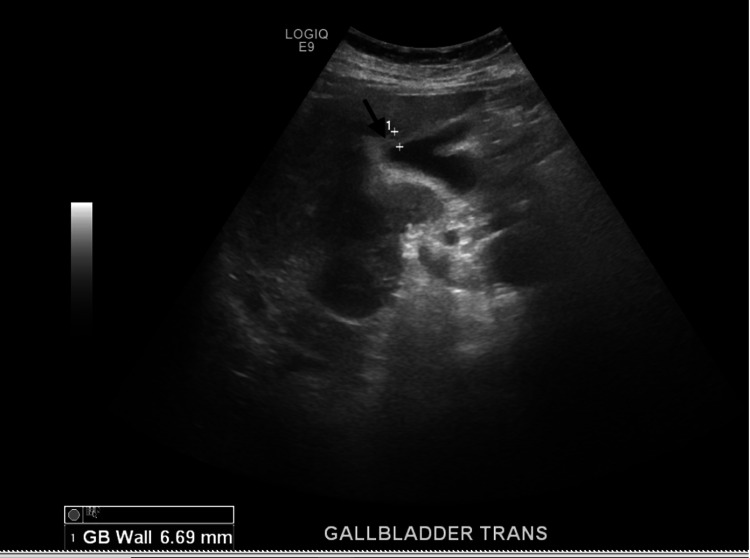
Ultrasound of gallbladder: arrow showing gallbladder wall thickness

Upon initial evaluation and physical exam, the patient was fully awake and oriented to person, place, and time. Initial vitals were as follows: respiratory rate of 23 breaths/min, oxygen saturation of 86% on room air which later improved to 94% with 2 L of oxygen via nasal cannula, blood pressure of 127/87 mmHg, pulse rate of 89 bpm, and temperature of 98.6°F. Head, eyes, ears, nose, and throat (HEENT) exam revealed pink conjunctiva and nonicteric sclera, dry tongue, and buccal mucosa. On respiratory system exam, she had mild tachypnea with a respiratory rate of 23 breaths/min and bilateral coarse crackles with rhonchi. Heart sounds were irregularly irregular, S1 and S2 well heard no murmur or gallop. Jugular venous pressure (JVP) was flat, and no distended neck veins. The abdomen was slightly distended abdomen which moved with respiration. The patient had marked right upper quadrant tenderness on both superficial and deep palpation with significant guarding and rigidity, and murphy’s sign was positive. She had no costovertebral angle tenderness or suprapubic tenderness. Extremity exam revealed mild pretibial and pedal trace edema. Laboratory tests revealed a white blood cell (WBC) count of 5,800 k/uL, hemoglobin of 13.2 g/dL, platelet of 170,000 k/uL, blood urea nitrogen (BUN) of 17 mg/dL, creatinine of 0.7 mg/dL, brain natriuretic peptide (BNP) of 398 pg/mL,aspartate transaminase (AST) of 21 u/L, alanine transaminase (ALT) of 14 u/L, alkaline phosphatase 62 u/L, and total bilirubin of 1.4 mg/dL. Inflammatory markers were elevated with C-reactive protein (CRP) of 68 mg/dL, lactate dehydrogenase (LDH) of 278 u/L, and D-dimer of 0.77 mg/L FEU (fibrinogen equivalent units)​​​​​​. ​Procalcitonin was within normal limits (0.20 ug/L). Blood cultures showed no growth in two bottles. COVID-19 nasal swab test was positive. Chest X-ray showed bilateral interstitial infiltrates typical of viral interstitial pneumonia (Figure 1). Ultrasound of the gallbladder showed gallbladder wall thickness with mild pericholecystic fluid collection and no gallstone suggestive of acalculous acute cholecystitis (Figures 2, 3). The most recent echocardiogram showed a left ventricular ejection fraction (LVEF) of 50%.

The patient was admitted to the COVID-19 unit with the diagnosis of acute hypoxic respiratory failure due to COVID-19 pneumonia and acute acalculous cholecystitis. She was treated with dexamethasone, remdesivir, and vitamin supplements. The patient also received supportive care with oxygenation via nasal cannula. Anticoagulation with Eliquis from her home medication was continued for the atrial fibrillation and deep vein thrombosis (DVT) prophylaxis. Empiric antibiotic coverage was initiated for acalculous cholecystitis with Zosyn after evaluation by the general surgeon. The patient was kept on nothing by mouth, and cholecystectomy was postponed as the patient clinically improved with empiric antibiotic coverage. The patient completed a 5-day course of remdesivir as an inpatient and eventually weaned off oxygen. Abdominal pain was significantly resolved, and oral feeding was resumed on the third day of her admission. Eventually, she was discharged home with oral Augmentin and the remaining dose of dexamethasone as well as vitamin supplements. She was given a follow-up appointment with general surgery as an outpatient for elective cholecystectomy after six weeks.

## Discussion

Our patient presented with the chief complaint of severe right upper quadrant abdominal pain and was found to have acute acalculous cholecystitis, which was caused by COVID-19 infection. The patient also had associated symptoms of COVID-19, such as dry cough and shortness of breath; however, they were not the primary reason for presenting to the hospital. Although in COVID-19, gastrointestinal (GI) involvement and particularly liver manifestation is well known, pure acalculous cholecystitis is extremely rare as an initial presenting symptom. On reviewing the literature, we found only two case reports of COVID-19 infection presenting with acalculous cholecystitis being the initial presenting symptoms [3].

The exact pathogenesis of acute acalculous cholecystitis in COVID-19 infection is not clear; however, it is proven that severe acute respiratory syndrome (SARS) coronaviruses have a tropism not only to the lungs but also to the liver. Intracellular entry of the virus occurs via an interaction with the angiotensin-converting enzyme 2 receptor (ACE2), which is present in several tissues, including lungs, liver, and biliary ducts. In SARS-CoV autopsies, liver tissue exhibited different patterns of hepatocyte injuries, and viral ribonucleic acid (RNA) was found inside hepatocytes [4]. That is how SARS-CoV-2 causes cytolysis with mild transaminase elevations in most cases [5,6]. Also, ACE2 is found in abundance on the epithelium of the gallbladder; hence, it could be a target for SARS-CoV-2 infection [7]. In two of the previously published cases of COVID-19 cholecystitis in whom emergency gall bladder surgery was done, even though reverse transcription-polymerase chain reaction (RT-PCR) of the bile did not detect the virus, quantitative RT-PCR (qRT-PCR) testing from the bile of these patients confirmed the presence of the virus, indicating that SARS-CoV-2 was specifically present in the gallbladder [8-10].

Our patient was treated non-operatively with antibiotics for the acalculous cholecystitis and improved; hence, we could not get the chance to do an RT-PCR test for COVID-19 from the bile. The patient was discharged with an appointment to get elective cholecystectomy after six to eight weeks by general surgery.

When SARS-CoV-2 is present uniquely with digestive symptoms without any respiratory manifestations, a longer time from the onset of symptoms and a worse prognosis is generally expected [11]. But this patient had both respiratory symptoms as well as GI symptoms; hence, the onset of the symptoms was not protracted.

This case presented with digestive symptoms preceding the classic respiratory manifestation of COVID-19. Her presentation exhibited an evolving clinical picture of acalculous cholecystitis with the radiological pattern of progressive gall bladder wall thickening, pericholecystic fluid collection, and a positive radiological and clinical Murphy sign. SARS-CoV-2 infection can cause acalculous acute cholecystitis.

## Conclusions

COVID 19 can rarely present with acute acalculous cholecystitis. This is suspected to be due to the tropism of the virus to tissues containing angiotensin-converting enzyme 2 receptor (ACE2), which is present in several tissues, including lungs, liver, and biliary ducts. When SARS-CoV-2 present uniquely with digestive symptoms without any respiratory manifestations, a longer time from onset of symptoms and a worse prognosis is generally expected. While differential diagnosis can be challenging, rapid testing for SARS-CoV-2 and potentially conservative management might make a difference to patient outcomes.
